# Transcriptomic Analysis Reveals the Protection of Astragaloside IV against Diabetic Nephropathy by Modulating Inflammation

**DOI:** 10.1155/2020/9542165

**Published:** 2020-08-12

**Authors:** Yudi Zhang, Chunhe Tao, Chen Xuan, Junyan Jiang, Wenfu Cao

**Affiliations:** ^1^College of Traditional Chinese Medicine, Chongqing Medical University, Chongqing 400016, China; ^2^Chongqing Key Laboratory of Traditional Chinese Medicine for Prevention and Cure of Metabolic Diseases, Chongqing 400016, China; ^3^Department of Chinese Traditional Medicine, The First Affiliated Hospital of Chongqing Medical University, Chongqing 400016, China

## Abstract

**Background:**

Diabetic nephropathy (DN) is one of the leading causes of end-stage kidney disease. Recently, there is no specific drug available to block the kidney damage. Astragaloside IV (AS-IV) is a major active component of *Astragalus membranaceus* (Fisch) Bge and has been demonstrated to benefit the kidney functions. This study explores the potential pharmacological action of AS-IV in DN of rats.

**Methods:**

Male Sprague-Dawley rats were fed with high-fat diet and injected with streptozotocin to induce diabetes. The diabetic rats were randomized and treated with vehicle or AS-IV (80 mg/kg) daily by gavage for 12 weeks as the DN or AS-IV group, respectively. The normal control rats were fed with normal chow and injected with vehicles (*n* = 8 per group). These rats were monitored for diabetes- and kidney function-related measures. The expression profiles of gene mRNA transcripts in the kidney tissues were analyzed by RNA-seq and quantitative RT-PCR. The levels of advanced glycation end products (AGEs), IL-1*β*, and IL-18 in the serum samples and kidney tissues were quantified by ELISA. The levels of collagen IV (COL-4) and fibronectin (FN) expression in kidney tissues were examined by immunohistochemistry and Western blot.

**Results:**

In comparison with the DN group, AS-IV treatment significantly reduced blood glucose levels, food and water consumption, 24 h urine, renal index values, 24 h urine total proteins, blood urea nitrogen (BUN) levels, and creatinine clearance rates (CCR), accompanied by minimizing the DN-induced early kidney damages, fibrosis, and microstructural changes. Furthermore, AS-IV treatment significantly modulated the DN-altered gene transcription profiles in the kidney of rats, particularly for inflammation-related genes, including the nucleotide-binding oligomerization domain-like receptor signaling, which was validated by quantitative RT-PCR. AS-IV treatment significantly decreased the levels of serum and kidney AGEs, IL-1*β*, and IL-18 expression and fibrosis indexes in the kidney of rats.

**Conclusion:**

AS-IV treatment ameliorated the severity of DN by inhibiting inflammation-related gene expression in the kidney of rats.

## 1. Introduction

Diabetic nephropathy (DN) is one of the most common and primary chronic complications of diabetes and is the most common cause of end-stage kidney disease [1, 2]. The incidence of diabetes is increasing and estimated to be about 592 million people by 2035 in the world [3]. Additionally, DN is a high-risk factor for the development of cardiovascular disease [4]. DN is clinically characterized by proteinuria and impaired kidney function in diabetic patients [5–7]. Pathologically, DN patients usually have their kidney hypertrophy, base membrane thickening, extracellular matrix deposition, glomerular sclerosis, and interstitial fibrosis [8]. Currently, there is no specifically effective therapy for control of the DN progression. Although DN patients can be managed by control of blood glucose and blood pressure, many patients eventually progress into renal failure [9]. Hence, understanding the pathogenic process of DN and development of new therapeutic reagents will be of high significance.

It has been thought that DN is attributed to many factors, including inflammation [10]. Astragaloside IV (AS-IV) is a main bioactive component of *Astragalus membranaceus* (Fisch) Bge (*Astragalus*), which has been widely used for treatment of DN in the clinic [11, 12]. Recent studies have shown that AS-IV has potent antidiabetes, anti-inflammatory, antioxidative, antifibrotic, antihypertensive, and myocardial protective activities [13–17]. Furthermore, AS-IV treatment can inhibit the pathogenic process of DN in rodents [17–24]. In addition, AS-IV treatment ameliorates endoplasmic reticulum stress in renal tubular epithelial cells and improves renal function and fibrosis in animal models of DN [22, 25–28]. However, the molecular mechanisms underlying the pharmacological action of AS-IV remain unclear.

This study employed a rat model of DN to explore the transcriptomic profiles in the kidney following AS-IV treatment. Our findings indicated that AS-IV treatment modulated inflammatory signal pathways, particularly for the nucleotide-binding oligomerization domain- (NOD-) like receptor (NLR) signaling in the kidney of DN rats.

## 2. Materials and Methods

### 2.1. Drug Preparation

AS-IV (molecular C_41_H_68_O_14_, weight 784.97, purity > 98%) was purchased from Nanjing Dilger Medical Technology (Nanjing, China). AS-IV was dissolved in 0.5% carboxymethyl cellulose sodium (CMC-Na+) in H_2_O.

### 2.2. Animals and a Rat Model of Early DN

Male Sprague-Dawley (SD) rats (4 weeks old) were obtained from Laboratory Animal Center, Chongqing Medical University, Chongqing, China. They were housed in a specific pathogen-free facility with a 12-hour (h) cycle of light/dark at 22°C and allowed to access food and water *ad libitum*.

A rat model of early DN was established using high-fat diet (HFD, D12451, Research Diets, USA) and a small dose of streptozotocin (STZ) [29, 30]. Briefly, the rats were randomized and fed with normal chow (NC, *n* = 8) or HFD for 4 weeks. The normal chow-fed and HFD-fed rats were injected intraperitoneally with vehicle 0.1 M sodium citrate (pH 4.5) and STZ (35 mg/kg, Beijing Solarbio Science & Technology, Beijing, China), respectively. Three days later, their blood glucose (BG) levels were monitored using ACCU-CHEK Performa (Roche Diabetes Care GmbH, Shanghai, China). Individual rats with two consecutive BG levels > 16.7 mM were diagnosed as having diabetes. Two weeks after STZ injection, the diabetic rats were randomized and administrated with vehicle CMC-Na+ or 80 mg/kg AS-IV by gavage daily for 12 weeks as the DN-model or AS-IV group (*n* = 8 per group). Their body weights were measured weekly, and their BG levels were tested biweekly. The experimental protocol was approved by the Animal Experiments Ethical Review Committee of Chongqing Medical University, Chongqing, China.

### 2.3. Biochemical Characteristics in Urine and Serum

At the end of the experiment, the 24 h urine samples of rats in individual metabolic cages were collected for the detection of urine total proteins. The amounts of food and water consumed by individual rats were assessed. After measurement of urine volumes and centrifugation, the urine samples were stored at -80°C. Subsequently, the blood samples of individual rats were collected for preparation of serum samples and the rats were sacrificed.

The levels of 24 h urine proteins, 24 h urine creatinine (UCR), serum creatinine (SCR), and blood urea nitrogen (BUN) were assessed using Quick Start™ Bradford 1x Dye Reagent (Bio-Rad Laboratories, USA) and specific kits (Nanjing Jiancheng Bioengineering Institute, Nanjing, China), according to the manufacturer's instructions. The endogenous creatinine clearance rate (CCR) of individual rats was used to evaluate glomerular filtration rate. After being adjusted by body weights, the CCR of each animal was calculated by the formula of CCR (mL/min/kg) = urinary  creatinine  (*μ*M)∗urine  volume  (mL/min)/(serum creatinine (*μ*M)∗weight (kg)).

### 2.4. Kidney Histology

Rat kidneys were collected immediately after being sacrificed. The cortex of the right kidneys from three rats in each group was dissected and stored in RNA store reagent (Tiangen Biotech, Beijing, China) at 4°C overnight and later stored in -80°C for RNA extraction and transcriptome sequencing. The remaining kidney tissues were fixed in 4% paraformaldehyde and paraffin-embedded. The tissue sections (4 *μ*m) were stained with hematoxylin and eosin (HE), Masson trichrome, and Periodic Acid-Schiff (PAS) and examined under a light microscope. At least 5 random positive areas in each section were photoimaged (magnification ×400) and analyzed using ImageJ (National Institutes of Health, USA).

### 2.5. Electron Microscopy

The microstructural and morphological features of renal cells, such as the foot process of podocytes in the glomeruli, were examined by transmission electron microscopy (TEM). The renal cortex tissues in each animal were fixed with 2.5% dialdehyde and embedded. The ultrathin tissue sections (70 nm) were stained with uranyl acetate and lead citrate and examined by TEM (JEM-1400PLUS, Japan).

### 2.6. Transcriptomics

The total RNA was extracted from each renal cortex tissue, and after qualification and quantification, their mRNAs were enriched by poly-T oligo-attached magnetic beads and fragmented using divalent cations in NEBNext First Strand Synthesis Reaction Buffer (5X), followed by purification. Subsequently, the cDNA was synthesized using random hexamer primer and M-MuLV Reverse Transcriptase (RNase H^−^), DNA Polymerase I, and RNase H. Sequencing libraries were prepared using NEBNext® Ultra™ RNA Library Prep Kit for Illumina® (NEB, USA), according to the manufacturer's instruction, and the library was assessed using Agilent 2100 bioanalyzer. Finally, the library was sequenced on an Illumina® NovaSeq platform [31] and yielded 150 bp paired-end reads. The generated raw reads were evaluated by the CASAVA base, and after removing adapter or ploy-N and low-quality reads, the remaining high quality reads were evaluated for their Q30, GC content, and alignment efficiency.

To more accurately evaluate the effect of AS-IV on gene expression in the kidney of DN rats, the differentially expressed genes (DEGs) were selected based on the following criteria: compared with the normal group, the DEGs with upregulated expression in the DN group, but downregulated in the AS-IV group, and those downregulated in the DN group, but upregulated expression in the AS-IV group. The selected DEGs were analyzed by gene clustering, Gene Ontology (GO), and Kyoto Encyclopedia of Genes and Genomes (KEGG) enrichment (http://www.genome.jp/kegg/). Meanwhile, the enriched genes in the NLR signal pathway with significant difference between the DN-model and AS-IV group were further enriched by Gene Set Enrichment Analysis (GSEA) tool (http://software.broadinstitute.org/gsea/index.jsp), including subtle expression changes [31]. Furthermore, the interesting genes included the intersection of DEGs and enrichment genes in the NLR signaling pathway.

### 2.7. Reverse Transcription and Quantitative Polymerase Chain Reaction (RT-qPCR)

Total RNA was extracted from the kidney tissues of individual rats using RNAIso Plus (Takara, Japan) and reversely transcribed into cDNA using the PrimeScript™ RT Reagent Kit with gDNA Eraser (Perfect Real Time) (Takara), according to the manufacturer's instructions. The relative levels of target gene mRNA transcripts to the control GAPDH were determined using UltraSYBR Mixture (Cwbiotech, Beijing, China) and the specific primers (Supplementary Table S1). PCR reactions were performed in duplicate at 95°C for 10 min and subjected to 39 cycles of 95°C for 10s, 60°C for 30s, and 72°C for 32 s. The data were analyzed by 2^-*ΔΔ*Ct^.

### 2.8. ELISA

The fresh kidney tissues were homogenized in PBS and centrifuged. After quantification of total proteins, the kidney tissue samples were used for measurement of advanced glycation end products (AGEs) and cytokines. The levels of serum and kidney AGEs in individual animals were analyzed by ELISA using Rat AGEs ELISA Kit (CUSABIO, Wuhan, China), according to the manufacturer's instructions. The levels of IL-1*β* and IL-18 in the serum samples and kidney tissues were quantified by ELISA using the commercially available kits (Boster Biological Technology, Wuhan, China), according to the manufacturer's instructions. The experimental and control samples were simultaneously tested in triplicate.

### 2.9. Immunohistochemistry

The paraffin-embedded kidney tissue sections (3 *μ*m) were deparaffinized, rehydrated, and subjected to antigen retrieval, followed by inactivation of endogenous peroxidase with 3% H_2_O_2_ in methanol. After being blocked with 3% bovine serum albumin, the sections were incubated overnight with anti-COL4, anti-FN, or isotype control (Proteintech, Wuhan, China, 1 : 500 for all) and bound antibodies were detected by horseradish peroxidase- (HRP-) labeled secondary antibodies. The specific signals were visualized with DAB reagent (Servicebio, China) and counterstained with hematoxylin. The sections were observed under a light microscope, and five high power images selected randomly from each section were evaluated. The stained signals were quantified for average integrated optical density of each visual positive field using Image-Pro Plus system (Media Cybernetics, Bethesda, MD, USA).

### 2.10. Western Blot

The impact of AS-IV on kidney fibrosis in DN rats was examined by Western blot assays. Briefly, renal tissues from individual rats were homogenized by RIPA buffer containing phosphatase and protease inhibitors and centrifuged. After quantifying the protein concentrations using BCA method, the tissue lysates were separated by SDS-PAGE on 8-10% gels and electrically transferred onto PVDF membrane. The membrane was sealed by 5% skimmed milk in TBST buffer and incubated overnight at 4°C with anti-COL4 (Abcam, UK, 1 : 1000), anti-FN (Proteintech, 1 : 1000), and GAPDH (Servicebio, China, 1 : 3000). The bound antibodies were detected with HRP-conjugated secondary antibodies and visualized using the enhanced chemiluminescent reagent. The data were quantitated by densitometry using ImageJ software.

### 2.11. Statistical Analysis

All data were analyzed for their distribution. The data with a normal distribution are expressed as mean ± standard deviation (SD) and analyzed by one-way ANOVA and post-LSD test or Dunnett's T3 test. The skewed data are presented as median with range or transformed to rank cases for normalization and statistically analyzed. All statistical analyses were performed using SPSS 21.0 program. Statistical significance was determined when a *p* value < 0.05. The graphs were generated using GraphPad Prism 5 (GraphPad Software, San Diego, CA, USA), Adobe Photoshop CS6 software (Adobe Systems, San Jose, CA, USA), and Adobe Illustrator CC2019 software (Adobe Systems).

## 3. Results

### 3.1. AS-IV Treatment Ameliorates Clinical Symptoms in DN Rats

**Figure 1:**
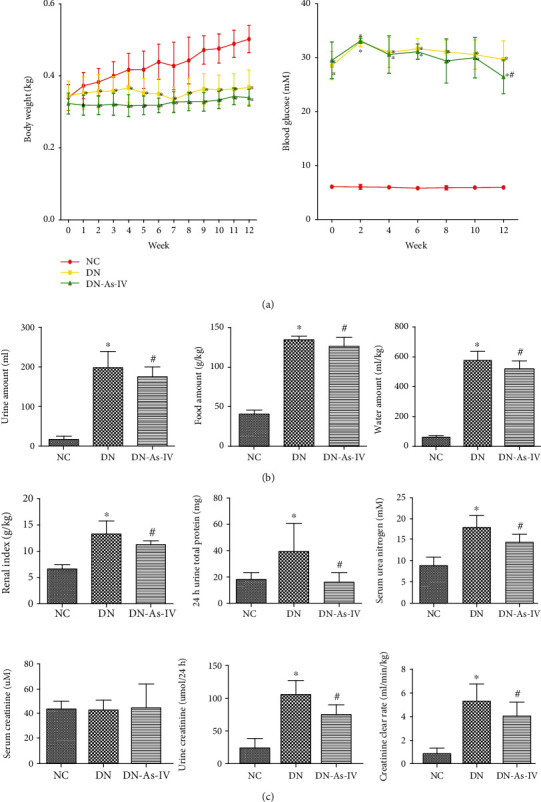
AS-IV treatment mitigates clinical symptoms and improves the kidney function in DN rats. Diabetic rats were randomized and treated with vehicle or AS-IV by gavage daily for 12 weeks and as the DN and AS-IV groups, respectively. One group of control (NC) received normal chow and vehicle treatment. (a) Their body weights and blood glucose levels were measured longitudinally. (b) Their water and food consumption and 24 h urine amounts were measured at the end of the experiment. (c) Their renal index, 24 h urine proteins, 24 h urine creatinine, blood urea nitrogen, serum creatinine levels, and creatinine clearance rate were measured. Data are expressed as the mean ± SD of each group (*n* = 8) of rats from three separate experiments, except for water, food, and urine amounts as the median and range. ^∗^*p* < 0.05 versus the NC group. ^#^*p* < 0.05 versus the DN group.

To understand the pharmacological action of AS-IV, a rat model of early DN was established and the diabetic rats were randomized and treated with vehicle or AS-IV for 12 weeks. To establish early DN, the rats were fed with HFD and injected with STZ. Throughout the 12 weeks, hyperglycemia gradually caused kidney damages in rats. In our preliminary study, we collected 24-hour urine every two weeks and measured the amount of urine total proteins. We found that the levels of 24-hour urine proteins in DN rats were >30 mg/24 h at least 8-10 weeks post hyperglycemia. Accordingly, we chose the 12-week time point in this study. The longitudinal measurements indicated that in comparison with that in the normal control (NC) group, the DN group of rats displayed significantly reduced body weights regardless of AS-IV treatment (*p* < 0.05 for both) and there was no significant difference in the body weights between the DN and AS-IV groups (Figure 1(a)). AS-IV treatment did significantly mitigate the DN-increased blood glucose in rats at 12-week posttreatment (*p* < 0.05, Figure 1(a)) although the levels of blood glucose in the AS-IV group of rats remained significantly higher than that in the NC group (*p* < 0.05, Figure 1(a)). Further analyses displayed that while the DN rats had significantly more water and food consumption and 24 h urine, relative to those in the NC group, AS-IV treatment significantly mitigated the amounts of water and food consumed and reduced levels of 24 h urine in rats (*p* < 0.05 for all, Figure 1(b)). Laboratory examinations revealed that the renal index values, the levels of 24 h urine total proteins, 24 h urine creatinine, BUN, and CCR in the AS-IV group were significantly higher than those in the NC group, but lower than those in the DN group of rats (*p* < 0.05 for all, Figure 1(c)). However, there was no significant difference in the levels of serum creatinine among these groups of rats. Collectively, such data indicated that the experimental protocol induced the early stage of renal injury and AS-IV treatment significantly ameliorated clinical symptoms and improved the kidney function in DN rats.

### 3.2. AS-IV Treatment Significantly Reduces the DN-Related Pathological Changes in the Kidney of Rats

**Figure 2:**
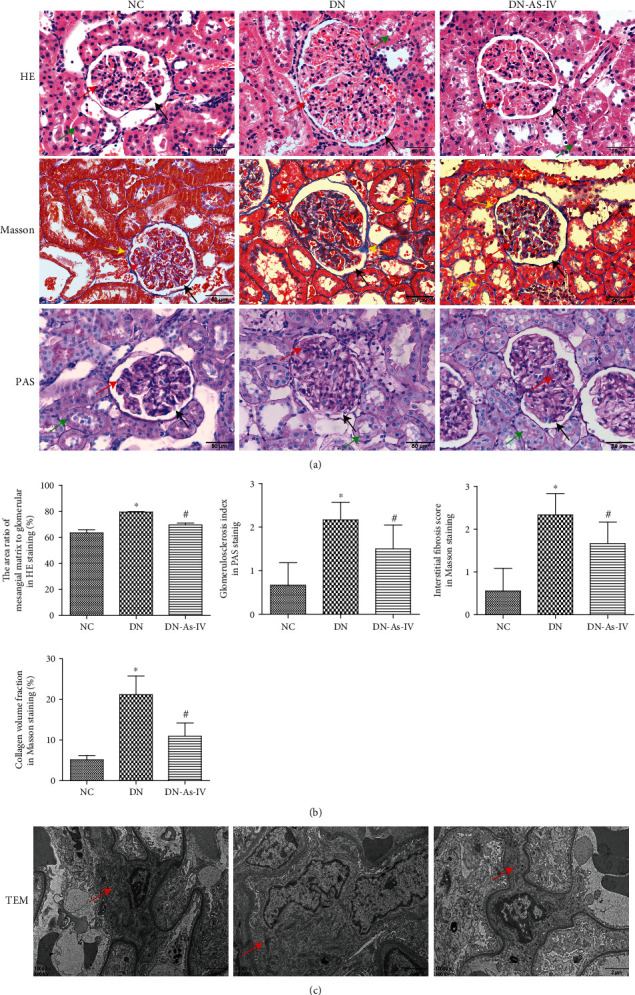
AS-IV treatment ameliorates the DN-related pathological changes in the kidney of rats. The kidney tissues were dissected from individual rats at the end of the experiment. The kidney tissue sections were stained with H&E, Masson, and PAS and subjected to TEM analysis, followed by semiquantitative analysis. Data are representative images or expressed as the mean ± SD of each group (*n* = 8) of rats from three separate experiments. (a) The H&E, Masson, and PAS (magnification ×400) analyses. (b) Semiquantitative analysis of the pathological changes in the kidney tissues. ^∗^*p* < 0.05 versus the NC group. ^#^*p* < 0.05 versus the DN group. (c) TEM (magnification ×10000) analyses. NC: normal control; DN: DN rats with vehicle treatment; DN-AS-IV: DN rats with AS-IV treatment. Black arrow: renal vesicle. Red arrow: mesangial region. Green arrow: renal tubular epithelial cells. Yellow arrow: basement membrane.

Next, we measured the histopathological changes in the kidney of different groups of rats after HE, Masson, and PAS staining and TEM analysis. In comparison with that in the NC group, the DN group of rats displayed severe pathological changes in glomerular, tubular, and renal interstitial morphology, such as glomerular hypertrophy, the increased thicknesses of glomerular basement membrane (GBM) and tubular basement membrane (TBM), mesangial expansion with increased matrix, podocyte damage, tubular epithelial cell vacuolar and granular degeneration, glomerular capsule cavity stenosis, and renal interstitial fibrosis, which were obviously reduced in the AS-IV group (Figure 2(a)). Quantitative analyses [16, 20, 24] revealed that AS-IV treatment significantly mitigated the DN-increased areas of mesangial matrix in the glomeruli and reduced the glomerulosclerosis index, interstitial fibrosis scores, and the percentages of collagen volume in the glomeruli of DN rats although they remained significantly higher than those in the NC group (*p* < 0.05 for all, Figure 2(b)). Furthermore, TEM analysis exhibited that compared with the NC group, the kidney from the DN group of rats displayed obviously increased thicknesses of the GBM, mild mesangial expansion, and local foot process fusion, which were less in the AS-IV group of rats (Figure 2(a)). These further indicated that the experimental protocol induced early stage of DN in rats. Together, AS-IV treatment significantly reduced the pathological changes, glycogen, and mucosubstance accumulation as well as fibrosis in the kidneys of DN rats.

### 3.3. AS-IV Treatment Modulates the Transcriptional Profiles in the Kidney of DN Rats

To understand the pharmacological action of AS-IV, we characterized the transcriptional profiles in the kidneys of different groups of rats. After extraction of total RNAs and enrichment of mRNAs, the mRNAs were reversely transcribed into cDNA to generate libraries, which were sequenced. Subsequently, the generated raw reads were evaluated by the CASAVA base and after removing adapter or ploy-N and low-quality reads, the remaining about 10.64 GB high-quality reads were evaluated for their Q30, GC content, and alignment efficiency. The sequenced reads had the alignment efficacies of 96.31%-96.9% (Supplementary Table 2).

**Figure 3:**
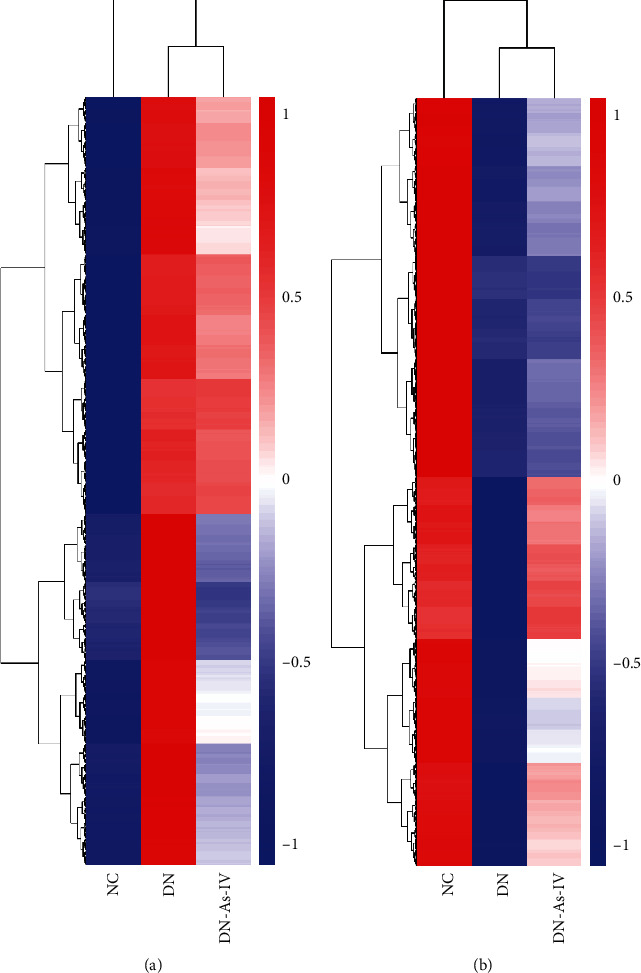
Heatmap analysis of DEGs among the kidneys of different groups of rats. At the end of treatment, three kidney cortex tissues from each group of rats were obtained and their RNAs were extracted for RNA-seq. The DEGs in the kidney tissues were hierarchically clustered and presented as heatmaps. (a) The downregulated DEGs (*n* = 3796) in the AS-IV group. (b) The upregulated DEGs (*n* = 3452) in the AS-IV group. NC: normal group; DN: diabetic nephropathy model group; DN-AS-IV: AS-IV-treatment group. Here, 1 represents the highest expression while -1 the lowest expression.

**Figure 4:**
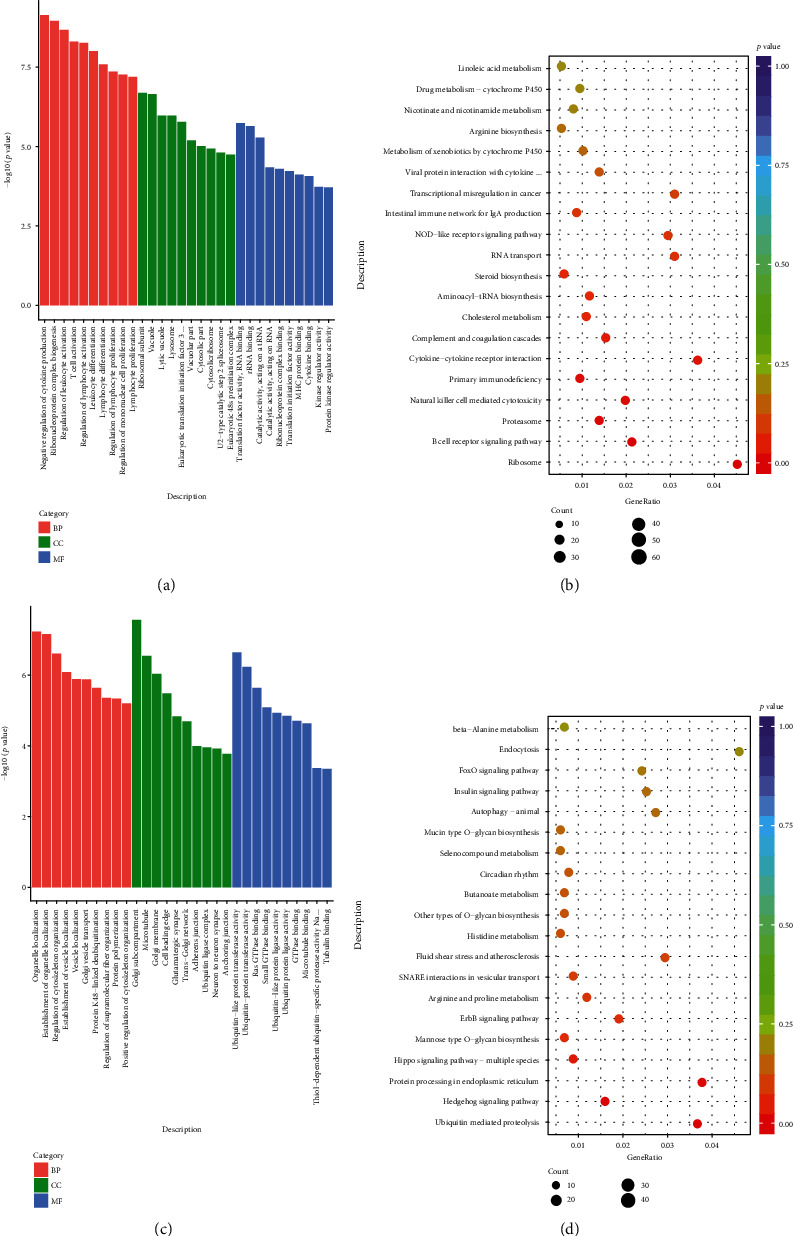
Functional and pathway enrichment analysis of DEGs among the different groups of rats: (a) GO enrichment analysis of downregulated DEGs in the AS-IV group of rats compared with DN rats; (b) KEGG enrichment analysis of downregulated DEGs in the AS-IV rats compared with DN rats; (c) GO enrichment analysis of upregulated DEGs in the AS-IV rats compared with DN rats; (d) KEGG enrichment analysis of upregulated DEGs in the AS-IV rats compared with DN rats. GO results were categorized into three parts: biological process (red), cellular component (green), and molecular function (blue). KEGG results were categorized into 20 main KEGG categories: weight greater, circle area larger.

Analysis of DEGs indicated that in comparison with the DN group, 3796 genes were downregulated while 3452 genes were upregulated in the AS-IV group of rats (Figure 3). GO enrichment analysis revealed that the 3796 downregulated DEGs were involved in the biological processes, such as regulation of cytokine production, inflammation, cell activation and proliferation, and T cell activation; cell components, like ribosomal subunit, lysosome, vacuolar part, and cytosolic part; and molecular functions, including translation factor activity, catalytic activity, MHC protein binding, cytokine binding, kinase regulator activity, and protein kinase regulator activity (Figure 4(a)). The KEGG pathway enrichment analyses indicated that these DEGs were mainly involved in immune and inflammatory responses, including the B cell receptor signaling, natural killer cell-mediated cytotoxicity, complement and coagulation cascades, and the NLR signaling (Figure 4(b)). Regulation of kidney metabolism was closely related to the cholesterol metabolism, metabolism of xenobiotics by cytochrome P450, arginine biosynthesis, nicotinate and nicotinamide metabolism, and others.

In contrast, the 3452 upregulated DEGs were involved in the biological process, such as organelle location, regulation of cytoskeleton organization, and vesicle location; cell components, like Golgi subcompartment, adherence junction, and ubiquitin ligase complex; and molecular functions, including ubiquitin-like protein transferase activity, GTPase binding, microtubule binding, and thiol-dependent ubiquitin-specific protease activity (Figure 4(c)). The KEGG pathway enrichment analyses indicated that these DEGs were closely related to the ubiquitin-mediated proteolysis, endoplasmic reticulum, autophagy, insulin signal pathway, and others in the kidneys of rats (Figure 4(d)).

### 3.4. AS-IV Treatment Modulates the NLR Signaling in the Kidney of DN Rats

**Figure 5:**
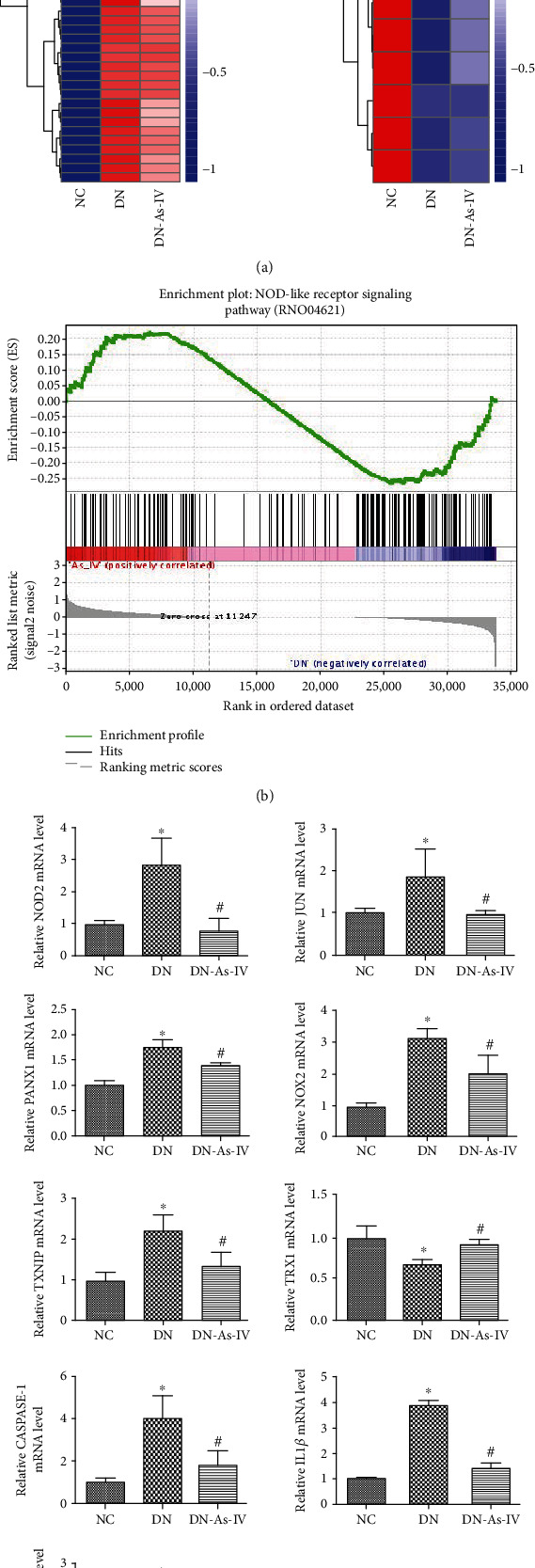
AS-IV treatment mitigates the expression of NLR signal events in the kidney of DN rats. GSEA analysis and quantification of interesting genes in the NOD-like receptor signaling pathway to compare DN with DN-As-IV rats. NC: normal group; DN: diabetic nephropathy model group; DN-AS-IV: AS-IV-treatment group. (a) The DEGs in the intersection of DEGs and NLR signaling-related genes among the DN, NC, and AS-IV groups of rats were hierarchically clustered and presented as heatmaps. There were 46 downregulated DEGs and 13 upregulated in the AS-IV group compared with the DN group. (b) The enriched genes in the NLR signaling pathway between the DN and AS-IV groups of rats were analyzed by GSEA method. There were 63 gene expression significantly enriched in the DN group, related to that in the AS-IV group. (c) Quantitative PCR analysis of the interesting genes. We found that AS-IV treatment mitigated the DN-modulated NOD2, JUN, PANX1, NOX2, TXNIP, CASPASE-1, IL-1*β*, and IL-18 and increased TRX1 mRNA transcription in DN rats. Results are presented as the mean ± SD of each group from three separate experiments, except for IL-1*β* as the median and range. ^∗^*p* < 0.05 versus the NC group. ^#^*p* < 0.05 versus the DN group.

It is well known that the NLR signaling is the intracellular sensors of pathogen-associated molecular patterns (PAMPs) of the pattern recognition receptor (PRR) family. The NLR signaling can crosstalk with the TLR to activate NF-*κ*B and MAPK, leading to proinflammatory cytokine production. Given that AS-IV treatment ameliorated DN, we analyzed the expression profile of the NLR signal pathway by GSEA. As shown in Figure 5(a), the clustering of genes from the intersection of DEGs and NLR signaling-related genes revealed that the expression of 46 genes was downregulated while 13 were upregulated in the AS-IV group compared with the DN group. As shown in Figure 5(b), the expression of 63 genes was significantly enriched in the DN group, related to that in the AS-IV group. These interesting genes included NOD-containing 2 (NOD2), JUN, NADPH oxidase 2 (NOX2), thioredoxin (TRX1), TRX-interacting protein (TXNIP), pannexin1 (PANX1), Caspase1, interleukin- (IL-) 1*β*, and IL-18. Further qPCR revealed that compared with the NC group, significantly increased NOD2, JUN, PANX1, NOX2, TXNIP, Caspase1, IL-1*β*, and IL-18 and decreased TRX1 mRNA transcripts were detected in the DN group, which were significantly mitigated by AS-IV treatment in the kidney of DN rats (*p* < 0.05 for all, Figure 5(c)). Therefore, AS-IV treatment significantly mitigated the DN-modulated NLR signaling in the kidney of rats.

### 3.5. AS-IV Treatment Reduces AGEs and Inflammatory Cytokines in DN Rats

**Figure 6:**
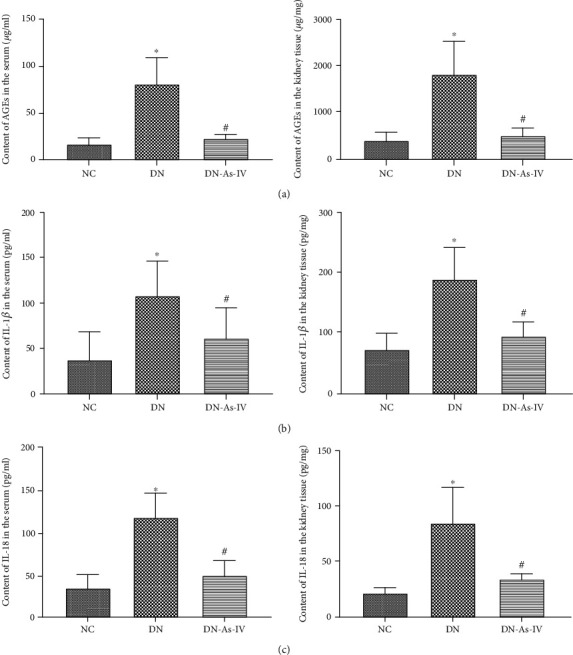
AS-IV treatment reduces the levels of AGEs, IL-1*β* and IL-18 in DN rats. The levels of AGEs, IL-1*β* and IL-18 in serum samples and kidney tissues from individual rats were measured by ELISA assays. (a) The levels of AGEs in the serum samples and kidney tissues from individual groups of rats. (b) The levels of IL-1*β* in the serum samples and kidney tissues from individual groups of rats. (c) The levels of IL-18 in the serum samples and kidney tissues from individual groups of rats. Data are presented as the mean ± SD of each group (*n* = 8-10) from three separate experiments. ^∗^*p* < 0.05 vs. the NC group. ^#^*p* < 0.05 vs. the DN group.

AGEs are kinds of senescent macroprotein derivatives formed at an accelerated rate under diabetes. AGEs through their signal-transducing receptor evokes oxidative stress and inflammation, thereby contributing to the pathogenesis of DN [32–34]. Meanwhile, we measured the levels of serum and kidney AGEs in individual rats by ELISA. As shown in Figure 6(a), AS-IV decreased the levels of serum and kidney AGEs in the DN rats, relative to those in the untreated DN rats (*p* < 0.05). These results revealed that AS-IV decreased AGE responses in DN rats.

IL-1*β* and IL-18 are the most important inflammatory cytokines in the downstream of the NLR signal pathway [35]. Their levels are very important for the function of kidney cells during the process of DN. Accordingly, we measured the levels of IL-1*β* and IL-18 in the serum samples and kidney tissues by ELISA. As shown in Figures 6(b) and 6(c), AS-IV decreased the levels of IL-1*β* and IL-18 in the serum samples and kidney tissues from DN rats, relative to those in the DN rats (*p* < 0.05 for all). These results revealed that AS-IV could relieve the expression of IL-1*β* and IL-18 in DN rats.

### 3.6. AS-IV Treatment Minimizes Fibrosis in the Kidney of DN Rats

**Figure 7:**
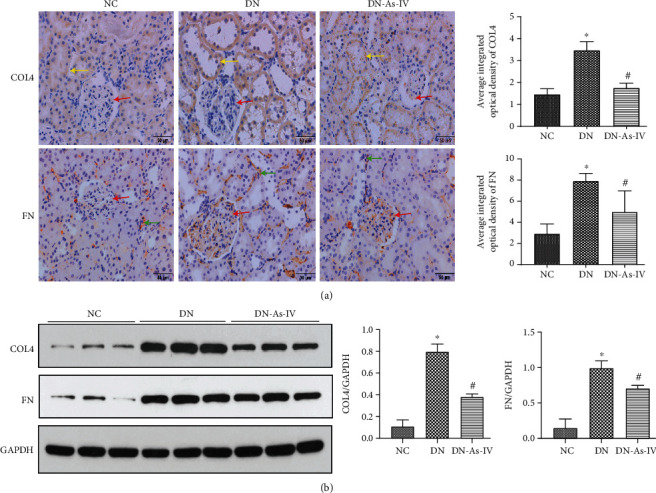
AS-IV treatment minimizes the COL4 and FN deposition in the kidney of DN rats. The levels of COL4 and FN in the kidney from individual rats were tested by immunohistochemistry and Western blot. (a) Immunohistochemistry analysis of the levels of COL4 and FN in the kidney of individual rats using specific anti-COL4 or anti-FN. (b) Western blot analysis of the levels of COL4 and FN in the kidney of individual rats. Data are representative images (magnification ×400) or expressed as the mean ± SD of each group (*n* = 8) from three separate experiments. ^∗^*p* < 0.05 vs. the NC group. ^#^*p* < 0.05 vs. the DN group. Red arrow: mesangial region. Green arrow: renal interstitium. Yellow arrow: basement membrane.

Renal fibrosis is a typical pathological change in the pathogenic process of DN [36]. Finally, we characterized the expression of COL4 and FN in the kidneys of different groups of rats. Immunohistochemistry displayed that in comparison with normal groups, the COL4 deposition increased mainly in the basement membrane in DN rats and the FN deposition increased mainly in the mesangial area and renal interstitium in DN rats. However, compared with that in the DN rats, AS-IV treatment decreased the COL4 and FN deposition in the basement membrane or interstitium or mesangial area in the kidney of DN rats (Figure 7(a)). Further Western blot analysis revealed that AS-IV reduced the relative levels of COL4 and FN expression in the kidney tissues from DN rats (*p* < 0.05 for all, Figure 7(b)). These results revealed that AS-IV decreased the expression and deposition of extracellular matrix, delaying the process of renal fibrosis in DN rats.

## 4. Discussion

In the book *Yellow Emperor's Internal Classic*, diabetes is described as “xiao-ke”—“a disease with symptoms of frequent drinking, diet, urination and weigh loss” and DN belongs to edema, urine turbidity, and kidney labor. DN is attributed to the poor control of thirst, which gradually injures the five organs (heart, liver, spleen, lung, and kidney), especially the kidney. Pathologically, DN is characterized by interlocking and accumulation of phlegm and blood stasis, its pathogenesis is Qi-deficiency, phlegm stagnation, and blood stasis. Among them, Qi deficiency is the most important pathogenesis. Astragalus membranaceus (Fisch) Bge is the most important Chinese medicine for Qi-tonifying and frequently prescribed for DN patients [11, 12]. Astragalus membranaceus (Fisch) Bge has been made as tablets for convenient application. AS-IV is a main bioactive component in Astragalus membranaceus (Fisch) Bge [37]. AS-IV has potent antidiabetes, antioxidation, anti-inflammatory, antifibrosis, and nephroprotective activities during the process of DN [17].

Hyperglycemia can induce excessive AGE formation, oxidative stress, damaged mitochondria accumulation, and increased ROS production in mesangial cells and reduce podocyte adhesion [38–41]. In this study, we explored the pharmacological action of AS-IV in a rat model of DN and found that treatment with AS-IV for 12 weeks significantly reduced food and water consumption, blood glucose levels, and urine amounts in rats, consistent with improved clinical symptoms [40]. Furthermore, treatment with AS-IV significantly mitigated the DN-related kidney GBM and TBM thicknesses, podocyte injury, and collagen deposition, the hallmarks of DN-related early kidney damages [40, 42]. As a result, AS-IV treatment significantly improved the kidney function by reducing BUN levels, 24 h urine creatinine, and proteinuria in early DN rats. Furthermore, we did not detect significantly higher SCR in the DN group of rats, inconsistent with previous observations [26]. However, we detected significantly higher CCR in the DN group, which was reduced in the AS-IV group. During the process of DN, several factors induce renal damages, which gradually display glomerular hyperfiltration, albuminuria, declined glomerular filtration rate (GFR) and, finally end-stage renal disease [6]. In the early stage of DN, glomerular hyperfiltration results in a higher GFR [6]. With the disease progression, increased proteinuria decreases the GFR [6]. The levels of kidney function and pathological changes suggest that our experimental protocol induced the early stage of DN in rats. These findings extended previous observations [26, 43] and support the notion that AS-IV treatment ameliorates the progression of DN and improves the kidney function.

Previous studies have shown that oxidative stress and inflammation are crucial for the development of DN [44, 45]. In this study, we characterized the mRNA transcription profiles in the renal cortex of the different groups of rats. We found that AS-IV treatment significantly altered the transcription of many genes that were involved in the biological processes, cellular components, and molecular functions, particularly for inflammatory responses. These indicated that inflammation contributed to the progression of DN in rats. The KEGG analyses revealed that AS-IV treatment altered various pathways, including immune and inflammation response and metabolic, ubiquitination, endoplasmic reticulum, and autophagy pathways, supporting the previous reports [16, 46]. Such novel findings may provide new insights into the pharmacological action of AS-IV in regulating the pathogenesis of DN in rats.

Hyperglycemia is usually accompanied by an accelerated rate of AGE formation. The serum AGE levels were significantly increased in diabetic patients [47, 48]. Accumulation of AGEs in the kidney may contribute to the progressive alteration in renal architecture and loss of renal function via various mechanisms [49–52], contributing to basement membrane thickening and mesangial expansion, hallmarks of DN [53, 54]. Our results demonstrated that AS-IV treatment significantly reduced the levels of serum and kidney AGEs and improved the pathological structure and function of kidney in DN rats, consistent with a previous report [55].

Oxidative stress is one of the factors to induce renal injury, contributing to the pathogenesis of DN [56]. Hyperglycemia can directly cause oxidative stress by promoting the production of ROS, and AGEs can cause renal damage through oxidative stress during the process of DN [57–59]. Studies have shown that excessive ROS can promote the production of cytokines and transcription factors, ultimately leading to the synthesis and deposition of extracellular matrix and promoting the progression of renal fibrosis and end-stage renal failure in the early and late stages of DN [60, 61]. The thioredoxin (TRX) system plays an important role in defending against oxidative stress [62] and is closely related to the NLRP3 inflammasome signaling pathway [63]. The TRX system consists of NADPH, TRXR, TRX, and TXNIP. TXNIP, also known as VDUP1, is the only endogenous negative regulator of TRX. TXNIP can not only block the antioxidant function of TRX but also induce the NLRP3 activation [64]. Our experiment results indicated that AS-IV treatment significantly decreased the relative levels of TXNIP but upregulated TRX1 mRNA transcripts in the kidney of DN rats. These results indicated that AS-IV could protect the kidney by enhancing the TRX antioxidant system.

Inflammatory cytokines are crucial for the development of DN [45]. The NLR signaling is closely related to the pathogenic infection and subsequent inflammation and immune responses [65]. The NLR family currently has 22 members, which is mainly divided into four subfamilies, based on the N-terminal domain. Among these members, NOD1 and NOD2 in the NLRC subfamily can activate the NF-*κ*B and MAPK signal pathways [66]. Other important members consist mainly of intracellular receptors, such as the NLRP subfamily member, ASC, and procaspase-1. Functionally, the NLR signaling can activate procaspase-1, leading to the production of inflammatory factors, including IL-1*β* and IL-18 [67]. NOD2 is an important factor and widely expressed by various types of cells [68]. A recent study has shown that NOD2 is highly expressed in kidney tissues [69] and contributes to the pathogenesis of DN [70]. IL-1*β* and IL-18 are the most important inflammatory cytokines in the downstream of the NLR signaling [35]. Their levels in the kidney are very important for the function of kidney cells during the process of DN. In our study, we found that AS-IV treatment significantly decreased the relative levels of NOD-2, JUN, NOX2, PANX1, TXNIP, caspase1, IL-1*β*, and IL-18 but upregulated TRX1 mRNA transcripts in the kidney of DN rats. Furthermore, AS-IV treatment significantly decreased the levels of IL-1*β* and IL-18 in serum samples and kidney tissues of DN rats. It is well known that c-JUN is a key factor of the AP-1 early response transcription factor in the MAPK signaling [71] and is associated with the development of diabetes and its microvascular complications [72, 73]. NOX is critical for the production of reactive oxygen species (ROS), leading to oxidative stress that contributes to DN development [50, 51] [52, 53]. Furthermore, high glucose can induce NOX2 expression, leading to oxidative stress in renal tubular epithelial and glomerular mesangial cells [74, 75]. TXNIP acts as an endogenous TRX inhibitor that reduces TRX protein activity and expression [76]. Aberrant oxidative stress and high levels of ROS can activate TXNIP by releasing TXNIP from the TXNIP/TRX complex, which subsequently leads to the NLRP3 activation [64, 77, 78]. PANX1 is a member of the innexin family, and functionally, it regulates the gap junctions [79, 80]. PANX1 can stimulate the ROS production and inflammasome activation, leading to caspase1 and IL-1*β* activation [81, 82]. Our data suggest that AS-IV may ameliorate hyperglycemia and inhibit the NOD2-related NF-*κ*B signaling, which crosstalks with the MAPK signaling, attenuating inflammation in DN rats. Simultaneously, AS-IV may suppress NOX2 expression, together with inhibition of PANX1 expression, to reduce ROS production and ROS-related TXNIP-promoted NLRP3 activation by enhancing TRX expression, inhibiting caspase1 activation, and IL-1*β* and IL-18 production in the kidney of DN rats [52, 54]. Excessive AGE formation, oxidative stress and inflammatory responses can aggravate the progression of renal fibrosis and promote the deposition of extracellular matrix in the kidney of DN rats [36, 83]. COL4 and FN are the main components of extracellular matrix [84]. Our experiment results revealed that AS-IV treatment significantly reduced the expression and deposition of extracellular matrix components in the kidney, delaying the process of renal fibrosis in DN rats. Hence, our novel findings may provide new insights into the pathogenesis of DN and the pharmacological action of AS-IV in inhibiting the progression of DN in rats. Conceivably, the hyperglycemia-related oxidative stress and inflammation to activate the NLR signaling may be new therapeutic targets for intervention of renal fibrosis in DN.

## 5. Conclusion

In summary, our data indicated that AS-IV treatment ameliorated clinical and pathological changes and improved the kidney function in DN rats. Transcriptomic analyses revealed that AS-IV treatment significantly mitigated the transcription of DN-related oxidative stress and inflammation-related NLR signal events in the kidney of rats. The decreased levels of AGEs, inflammation cytokines, and fibrosis further revealed that AS-IV treatment attenuated the NLR signaling and delayed the process of renal fibrosis in DN rats. Therefore, our findings may reveal new therapeutic targets for intervention of DN and provide new insights into the pharmacological action of AS-IV in inhibiting the progression of DN in rats.

## Data Availability

The raw data used to support the findings of this study are available from the first author and corresponding author upon request. The raw data mainly included transcriptome analysis, physiological and biochemical markers, histopathological picture, and gene verification.

## Supplementary Materials

Supplementary Materials Table S1: the sequences of primers. Table S2: quality control of sequencing data.

## References

[1] de Boer I. H., Rue T. C., Hall Y. N., Heagerty P. J., Weiss N. S., Himmelfarb J. (2011). Temporal trends in the prevalence of diabetic kidney disease in the United States. *JAMA*.

[2] Collins A. J., Foley R. N., Chavers B. (2012). US Renal Data System 2011 Annual Data Report. *American Journal of Kidney Diseases*.

[3] Guariguata L., Whiting D. R., Hambleton I., Beagley J., Linnenkamp U., Shaw J. E. (2014). Global estimates of diabetes prevalence for 2013 and projections for 2035. *Diabetes Research and Clinical Practice*.

[4] Pugliese G. (2014). Updating the natural history of diabetic nephropathy. *Acta Diabetologica*.

[5] Viberti G. C., Jarrett R. J., Keen H. (1982). MICROALBUMINURIA AS PREDICTOR OF NEPHROPATHY IN DIABETICS. *Lancet*.

[6] Mogensen C. E., Christensen C. K., Vittinghus E. (1983). The stages in diabetic renal disease. With emphasis on the stage of incipient diabetic nephropathy. *Diabetes*.

[7] Mogensen C. E., Christensen C. K. (1984). Predicting diabetic nephropathy in insulin-dependent patients. *The New England Journal of Medicine*.

[8] Najafian B., Alpers C. E., Fogo A. B. (2011). Pathology of human diabetic nephropathy. *Contributions to Nephrology*.

[9] Cooper M. E. (1998). Pathogenesis, prevention, and treatment of diabetic nephropathy. *Lancet*.

[10] Schmid H., Boucherot A., Yasuda Y. (2006). Modular activation of nuclear factor- B transcriptional programs in human diabetic nephropathy. *Diabetes*.

[11] Ai P., Yong G., Dingkun G., Qiuyu Z., Kaiyuan Z., Shanyan L. (2008). Aqueous extract of _Astragali Radix_ induces human natriuresis through enhancement of renal response to atrial natriuretic peptide. *Journal of Ethnopharmacology*.

[12] Yu L., Li J. Z., Wang H. Y. (2001). Progress in the study of the treatment of nephropathy with Astragalus and Angelica and their therapeutic mechanism. *Chinese Journal of Integrated Traditional and Western Medicine*.

[13] Luo H. M., Dai R. H., Li Y. (1995). Nuclear cardiology study on effective ingredients of Astragalus membranaceus in treating heart failure. *Zhongguo Zhong Xi Yi Jie He Za Zhi*.

[14] He Y., Du M., Gao Y. (2013). Astragaloside IV attenuates experimental autoimmune encephalomyelitis of mice by counteracting oxidative stress at multiple levels. *PLoS ONE*.

[15] Zhang N., Wang X. H., Mao S. L., Zhao F. (2011). Astragaloside IV improves metabolic syndrome and endothelium dysfunction in fructose-fed rats. *Molecules*.

[16] Gui D., Huang J., Guo Y. (2013). Astragaloside IV ameliorates renal injury in streptozotocin-induced diabetic rats through inhibiting NF-*κ*B-mediated inflammatory genes expression. *Cytokine*.

[17] Li L., Hou X., Xu R., Liu C., Tu M. (2017). Research review on the pharmacological effects of astragaloside IV. *Fundamental & Clinical Pharmacology*.

[18] Chen Y., Gui D., Chen J., He D., Luo Y., Wang N. (2014). Down-regulation of PERK-ATF4-CHOP pathway by Astragaloside IV is associated with the inhibition of endoplasmic reticulum stress-induced podocyte apoptosis in diabetic rats. *Cellular Physiology and Biochemistry*.

[19] Chen Q., Su Y., Ju Y., Ma K., Li W., Li W. (2018). Astragalosides IV protected the renal tubular epithelial cells from free fatty acids-induced injury by reducing oxidative stress and apoptosis. *Biomedicine & Pharmacotherapy*.

[20] Chen J., Chen Y., Luo Y., Gui D., Huang J., He D. (2014). Astragaloside IV ameliorates diabetic nephropathy involving protection of podocytes in streptozotocin induced diabetic rats. *European Journal of Pharmacology*.

[21] Chen J., Gui D., Chen Y., Mou L., Liu Y., Huang J. (2008). Astragaloside IV improves high glucose-induced podocyte adhesion dysfunction via *α*3*β*1 integrin upregulation and integrin-linked kinase inhibition. *Biochemical Pharmacology*.

[22] Wang X., Gao Y., Tian N., Zou D., Shi Y., Zhang N. (2018). Astragaloside IV improves renal function and fibrosis via inhibition of miR-21-induced podocyte dedifferentiation and mesangial cell activation in diabetic mice. *Drug Design, Development and Therapy*.

[23] Lu W. S., Li S., Guo W. W., Chen L. L., Li Y. S. (2015). Effects of Astragaloside IV on diabetic nephropathy in rats. *Genetics and Molecular Research*.

[24] Sun H., Wang W., Han P. (2016). Astragaloside IV ameliorates renal injury in db/db mice. *Scientific Reports*.

[25] Roglic G., World Health Organization (2016). *Global report on diabetes*.

[26] Ju Y., Su Y., Chen Q. (2019). Protective effects of Astragaloside IV on endoplasmic reticulum stress-induced renal tubular epithelial cells apoptosis in type 2 diabetic nephropathy rats. *Biomedicine & Pharmacotherapy*.

[27] Wang X., Gao Y., Tian N. (2018). Astragaloside IV represses high glucose-induced mesangial cells activation by enhancing autophagy via SIRT1 deacetylation of NF-kappaB p65 subunit. *Drug Design, Development and Therapy*.

[28] Liu X., Wang W., Song G. (2017). Astragaloside IV ameliorates diabetic nephropathy by modulating the mitochondrial quality control network. *PLoS One*.

[29] Hou B., Qiang G., Zhao Y. (2018). Salvianolic acid A protects against diabetic nephropathy through ameliorating glomerular endothelial dysfunction via inhibiting AGE-RAGE signaling. *Cellular Physiology and Biochemistry*.

[30] Dhar M. S., Sommardahl C. S., Kirkland T. (2004). Mice heterozygous for Atp10c, a putative amphipath, represent a novel model of obesity and type 2 diabetes. *The Journal of Nutrition*.

[31] Huang X., Yan J., Zhang M. (2018). Targeting epigenetic crosstalk as a therapeutic strategy for EZH2-aberrant solid tumors. *Cell*.

[32] Yamagishi S., Imaizumi T. (2005). Diabetic vascular complications: pathophysiology, biochemical basis and potential therapeutic strategy. *Current Pharmaceutical Design*.

[33] Yamagishi S., Matsui T. (2010). Advanced glycation end products, oxidative stress and diabetic nephropathy. *Oxidative Medicine and Cellular Longevity*.

[34] Wendt T., Bucciarelli L., Qu W. (2002). Receptor for advanced glycation endproducts (RAGE) and vascular inflammation: insights into the pathogenesis of macrovascular complications in diabetes. *Current Atherosclerosis Reports*.

[35] Hutton H. L., Ooi J. D., Holdsworth S. R., Kitching A. R. (2016). The NLRP3 inflammasome in kidney disease and autoimmunity. *Nephrology (Carlton)*.

[36] Brosius F. C. (2008). New insights into the mechanisms of fibrosis and sclerosis in diabetic nephropathy. *Reviews in Endocrine & Metabolic Disorders*.

[37] Qian W., Qian Q., Cai X. (2019). Astragaloside IV inhibits oxidized low-density lipoprotein-induced endothelial damage via upregulation of miR-140-3p. *International Journal of Molecular Medicine*.

[38] Nishikawa T., Edelstein D., du X. L. (2000). Normalizing mitochondrial superoxide production blocks three pathways of hyperglycaemic damage. *Nature*.

[39] Sagoo M. K., Gnudi L. (2018). Diabetic nephropathy: is there a role for oxidative stress?. *Free Radical Biology & Medicine*.

[40] Krata N., Zagożdżon R., Foroncewicz B., Mucha K. (2018). Oxidative stress in kidney diseases: the cause or the consequence?. *Archivum Immunologiae et Therapiae Experimentalis (Warsz)*.

[41] Forbes J. M., Thallas V., Thomas M. C. (2003). The breakdown of preexisting advanced glycation end products is associated with reduced renal fibrosis in experimental diabetes. *The FASEB Journal*.

[42] Miranda-Díaz A. G., Pazarín-Villaseñor L., Yanowsky-Escatell F. G., Andrade-Sierra J. (2016). Oxidative stress in diabetic nephropathy with early chronic kidney disease. *Journal Diabetes Research*.

[43] Li C., Liu X., Yong N. (2004). Therapeutic effect of curcumin on renal lesions and its influence on the expression of Smad7 in diabetic rats. *Acta Universitatis Medictnae Tangji*.

[44] Kandhare A. D., Mukherjee A., Bodhankar S. L. (2017). Antioxidant for treatment of diabetic nephropathy: a systematic review and meta-analysis. *Chemico-Biological Interactions*.

[45] Zheng S., Powell D. W., Zheng F., Kantharidis P., Gnudi L. (2016). Diabetic nephropathy: proteinuria, inflammation, and fibrosis. *Journal Diabetes Research*.

[46] Guo H., Cao A., Chu S. (2016). Astragaloside IV attenuates podocyte apoptosis mediated by endoplasmic reticulum stress through upregulating sarco/endoplasmic reticulum Ca2+-ATPase 2 expression in diabetic nephropathy. *Frontiers in Pharmacology*.

[47] Makita Z., Radoff S., Rayfield E. J. (1991). Advanced glycosylation end products in patients with diabetic nephropathy. *The New England Journal of Medicine*.

[48] Galler A., Muller G., Schinzel R., Kratzsch J., Kiess W., Munch G. (2003). Impact of metabolic control and serum lipids on the concentration of advanced glycation end products in the serum of children and adolescents with type 1 diabetes, as determined by fluorescence spectroscopy and nepsilon-(carboxymethyl)lysine ELISA. *Diabetes Care*.

[49] Bohlender J. M., Franke S., Stein G., Wolf G. (2005). Advanced glycation end products and the kidney. *American Journal of Physiology. Renal Physiology*.

[50] Beisswenger P. J., Makita Z., Curphey T. J. (1995). Formation of immunochemical advanced glycosylation end products precedes and correlates with early manifestations of renal and retinal disease in diabetes. *Diabetes*.

[51] Soulis-Liparota T., Cooper M., Papazoglou D., Clarke B., Jerums G. (1991). Retardation by aminoguanidine of development of albuminuria, mesangial expansion, and tissue fluorescence in streptozocin-induced diabetic rat. *Diabetes*.

[52] Bouma B., Kroon-Batenburg L. M. J., Wu Y. P. (2003). Glycation induces formation of amyloid cross-*β* structure in albumin. *The Journal of Biological Chemistry*.

[53] Mott J. D., Khalifah R. G., Nagase H., Shield C. F., Hudson J. K., Hudson B. G. (1997). Nonenzymatic glycation of type IV collagen and matrix metalloproteinase susceptibility. *Kidney International*.

[54] Brownlee M. (1994). Glycation and diabetic complications. *Diabetes*.

[55] Motomura K., Fujiwara Y., Kiyota N. (2009). Astragalosides isolated from the root of astragalus radix inhibit the formation of advanced glycation end products. *Journal of Agricultural and Food Chemistry*.

[56] Schiffer T. A., Friederich-Persson M. (2017). Mitochondrial reactive oxygen species and kidney hypoxia in the development of diabetic nephropathy. *Frontiers in Physiology*.

[57] Yamagishi S., Nakamura K., Matsui T. (2009). Regulation of advanced glycation end product (AGE)-receptor (RAGE) system by PPAR-gamma agonists and its implication in cardiovascular disease. *Pharmacological Research*.

[58] Pan H. Z., Zhang L., Guo M. Y. (2010). The oxidative stress status in diabetes mellitus and diabetic nephropathy. *Acta Diabetologica*.

[59] Reiniger N., Lau K., McCalla D. (2010). Deletion of the receptor for advanced glycation end products reduces glomerulosclerosis and preserves renal function in the diabetic OVE26 mouse. *Diabetes*.

[60] Chou S. T., Tseng S. T. (2017). Oxidative stress markers in type 2 diabetes patients with diabetic nephropathy. *Clinical and Experimental Nephrology*.

[61] Fernandes S. M., Cordeiro P. M., Watanabe M., Fonseca C. D., Vattimo M. F. F. (2016). The role of oxidative stress in streptozotocin-induced diabetic nephropathy in rats. *Archives of Endocrinology and Metabolism*.

[62] Yodoi J., Matsuo Y., Tian H., Masutani H., Inamoto T. (2017). Anti-inflammatory thioredoxin family proteins for medicare, healthcare and aging care. *Nutrients*.

[63] Zhu Y., Zhu C., Yang H., Deng J., Fan D. (2020). Protective effect of ginsenoside Rg5 against kidney injury via inhibition of NLRP3 inflammasome activation and the MAPK signaling pathway in high-fat diet/streptozotocin-induced diabetic mice. *Pharmacological Research*.

[64] Feng H., Gu J., Gou F. (2016). High glucose and lipopolysaccharide prime NLRP3 inflammasome via ROS/TXNIP pathway in mesangial cells. *Journal Diabetes Research*.

[65] Chen G., Shaw M. H., Kim Y. G., Nuñez G. (2009). NOD-like receptors: role in innate immunity and inflammatory disease. *Annual Review of Pathology*.

[66] Mukherjee T., Hovingh E. S., Foerster E. G., Abdel-Nour M., Philpott D. J., Girardin S. E. (2019). NOD1 and NOD2 in inflammation, immunity and disease. *Archives of Biochemistry and Biophysics*.

[67] Schroder K., Tschopp J. (2010). The inflammasomes. *Cell*.

[68] Zhao L., Hu P., Zhou Y., Purohit J., Hwang D. (2011). NOD1 activation induces proinflammatory gene expression and insulin resistance in 3T3-L1 adipocytes. *American Journal of Physiology. Endocrinology and Metabolism*.

[69] Lech M., Avila-Ferrufino A., Skuginna V., Susanti H. E., Anders H. J. (2010). Quantitative expression of RIG-like helicase, NOD-like receptor and inflammasome-related mRNAs in humans and mice. *International Immunology*.

[70] Du P., Fan B., Han H. (2013). NOD2 promotes renal injury by exacerbating inflammation and podocyte insulin resistance in diabetic nephropathy. *Kidney International*.

[71] Ozanne B. W., Spence H. J., McGarry L. C., Hennigan R. F. (2007). Transcription factors control invasion: AP-1 the first among equals. *Oncogene*.

[72] Wilmer W. A., Cosio F. G. (1998). DNA binding of activator protein-1 is increased in human mesangial cells cultured in high glucose concentrations. *Kidney International*.

[73] Tong Z., Yang Z., Patel S. (2008). Promoter polymorphism of the erythropoietin gene in severe diabetic eye and kidney complications. *Proceedings of the National Academy of Sciences of the United States of America*.

[74] Sedeek M., Nasrallah R., Touyz R. M., Hébert R. L. (2013). NADPH oxidases, reactive oxygen species, and the kidney: friend and foe. *Journal of the American Society of Nephrology*.

[75] Bai J., Wang Y., Zhu X., Shi J. (2019). Eriodictyol inhibits high glucose-induced extracellular matrix accumulation, oxidative stress, and inflammation in human glomerular mesangial cells. *Phytotherapy Research*.

[76] Nishiyama A., Matsui M., Iwata S. (1999). Identification of thioredoxin-binding protein-2/vitamin D3Up-regulated protein 1 as a negative regulator of thioredoxin function and expression. *The Journal of Biological Chemistry*.

[77] Bauernfeind F., Bartok E., Rieger A., Franchi L., Núñez G., Hornung V. (2011). Cutting edge: reactive oxygen species inhibitors block priming, but not activation, of the NLRP3 inflammasome. *Journal of Immunology*.

[78] Zhou R., Tardivel A., Thorens B., Choi I., Tschopp J. (2010). Thioredoxin-interacting protein links oxidative stress to inflammasome activation. *Nature Immunology*.

[79] Bruzzone R., Hormuzdi S. G., Barbe M. T., Herb A., Monyer H. (2011). Pannexins, a family of gap junction proteins expressed in brain. *Proceedings of the National Academy of Sciences of the United States of America*.

[80] Chen Y., Yao Y., Sumi Y. (2010). Purinergic signaling: a fundamental mechanism in neutrophil activation. *Science Signaling*.

[81] Hung S. C., Choi C. H., Said-Sadier N. (2013). P2X4 assembles with P2X7 and pannexin-1 in gingival epithelial cells and modulates ATP-induced reactive oxygen species production and inflammasome activation. *PLoS One*.

[82] Dahl G., Keane R. W. (2012). Pannexin: from discovery to bedside in 11±4 years?. *Brain Research*.

[83] Yamagishi S.-i., Takeuchi M., Makita Z. (2001). Advanced glycation end products and the pathogenesis of diabetic nephropathy. *Contributions to Nephrology*.

[84] Nam J. E., Jo S. Y., Ahn C. W., Kim Y. S. (2020). Baicalin attenuates fibrogenic process in human renal proximal tubular cells (HK-2) exposed to diabetic milieu. *Life Sciences*.

