# Supplementary material for: Transcriptomic Analysis Reveals the Protection of Astragaloside IV against Diabetic Nephropathy by Modulating Inflammation
Source: Oxid Med Cell Longev. 2020 Aug 12;2020:9542165. doi: 10.1155/2020/9542165 (PMC7443226; doi:10.1155/2020/9542165)
Supplement: Supplementary Materials — Table S1: the sequences of primers. Table S2: quality control of sequencing data. [file 9542165.f1.docx]

Table S1. **The sequences of primers**

| GAPDH Forward | TGTGACTTCAACAGCAACTCCC |
| --- | --- |
| Reward | CTCTCTTGCTCTCAGTATCCTTG |
| NOX2 Forward | TGCCAACTTCCTCAGCTACAAC |
| Reward | ATCTTTCTCCTCATCGTGGTG |
| TXNIP Forward | CACTGCGTTCTCTTGCAATCG |
| Reward | AAGAGCCTCCATCTATAACCC |
| CASPASE-1 Forward | GCAAGCCAGATGTTTATCACT |
| Reward | CGCCACCTTCTTTGTTCAGT |
| PANX1 Forward | TCAAGCCTGTGACTCCTGT |
| Reward | GCTATTCTTCCATAACGCTGT |
| IL-1β Forward | GGTACATCAGCACCTCTCAAGC |
| Reward | AAGTCAACTATGTCCCGACCA |
| IL-18 Forward | GACAAAAGAAACCCGCCTGT |
| Reward | AGCCAGTCCTCTTACTTCACT |
| JUN Forward | TCTCTCCCCAGCAACCCACA |
| Reward | TGGCTCTCAACTCAAGCGTCT |
| NOD2 Forward | TCGACCCCACGTCAGTCCA |
| Reward | ACAGTTTGATGCCCTCTTCGGA |
| TRX1 Forward | ATCAAGCCCTTCTTTCATTCCC |
| Reward | AACTCCCCAACCTTTTGACC |

Table S2. **Quality control of sequencing data**

| Group | NO. | Clean reads | Clean bases | GC% | ≥Q30% | alignment efficiency |
| --- | --- | --- | --- | --- | --- | --- |
| NC | N1 | 104667208 | 15.7G | 48.76 | 94.54 | 96.55% |
|  | N2 | 81947754 | 12.29G | 48.4 | 94.65 | 96.66% |
|  | N3 | 103975278 | 15.6G | 48.46 | 94.54 | 96.42% |
| DN | D1 | 81680268 | 12.25G | 48.16 | 94.41 | 96.67% |
|  | D2 | 116073508 | 17.41G | 48.44 | 94.6 | 96.5% |
|  | D3 | 84845236 | 12.73G | 47.67 | 94.79 | 96.9% |
| DN-As-Ⅳ | A1 | 70934568 | 10.64G | 48.14 | 94.31 | 96.45% |
|  | A2 | 90216290 | 13.53G | 48.66 | 94.37 | 96.31% |
|  | A3 | 100179088 | 15.03G | 48.37 | 94.28 | 96.31% |

Note: Clean reads [represents](http://dict.cnki.net/dict_result.aspx?searchword=%e4%bb%a3%e8%a1%a8&tjType=sentence&style=&t=represents) number of reads filtered from raw data; Clean bases means base filtered from raw data. The [content](http://dict.cnki.net/dict_result.aspx?searchword=%e5%90%ab%e9%87%8f&tjType=sentence&style=&t=content) of GC bases in the primer sequence is the percentage of total bases in clean data; ≥Q30 [represents](http://dict.cnki.net/dict_result.aspx?searchword=%e4%bb%a3%e8%a1%a8&tjType=sentence&style=&t=represents) the ratio of base with Phred value greater than 30% to total base in clean data.
